# Surgical approach in the management of self-inflicted penetrating abdominal trauma with knife-in-situ (Hunting Knife) in Vicente Sotto Memorial Medical Center: a tertiary institution in Cebu City, Philippines

**DOI:** 10.1136/tsaco-2024-001404

**Published:** 2024-04-24

**Authors:** Maymona Choudry, Stephen O Bullo, Clive Y Tabuena, Theo Genesis M Tagaytay

**Affiliations:** Department of General Surgery, Vicente Sotto Memorial Medical Center, Cebu City, Philippines

**Keywords:** Wounds, Penetrating, general surgery, laparotomy, abdominal injuries

## Surgical dilemma

**Figure 1:**
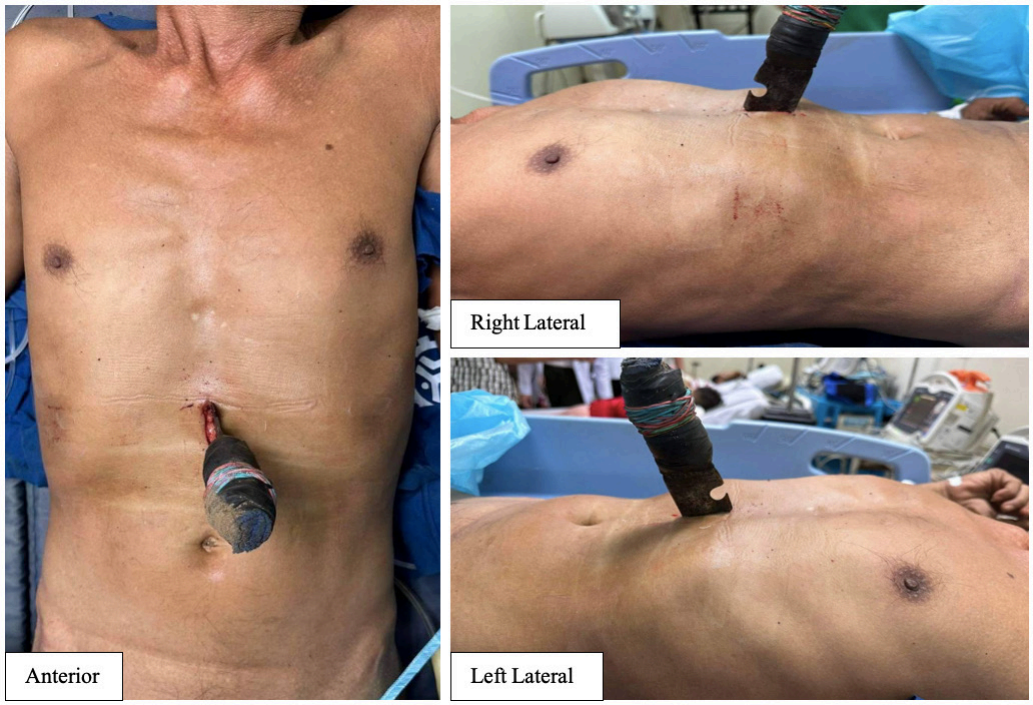
Patient came to the emergency room ~4 hours postinjury, with a penetrating abdominal trauma with a hunting knife-in-situ at epigastrium.

**Figure 2:**
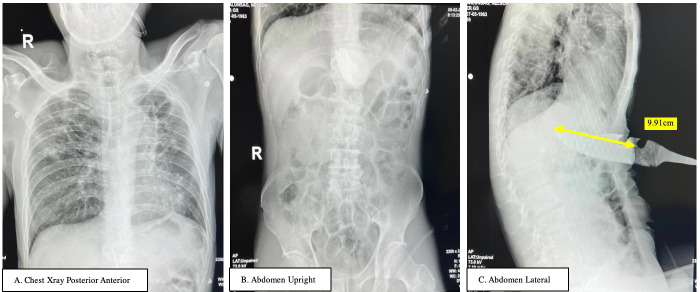
Chest (A) and abdominal X-rays (B,C) were taken, which showed that the depth of the hunting knife to be 9.91 cm (C).

This was a case of an adult patient in their late 50s, who came in ~4 hours post-injury with a self-inflicted stab wound at the epigastric region, with a hunting knife-in situ (figure 1). He was referred to Vicente Sotto Memorial Medical Center, a tertiary institution with a trauma center. At the emergency room, the patient arrived with stable vital signs, with a knife-in-situ at the epigastric region, but with no signs of acute abdomen. To avoid delays and stabilize the knife, only an abdominal X-ray was done to estimate the depth of the penetration. The abdominal series (figure 2) showed that the depth of the knife was 9.91 cm. The knife was stabilized to prevent any further injury.

## What would you do?

1. Perform simple extraction and wound exploration at the emergency room since the patient is hemodynamically stable and asymptomatic.
2. Perform laparotomy and extract the object in situ under direct visualization.
3. Perform a computed tomography scan of the abdomen with contrast since the patient is hemodynamically stable.

## What we did and why?

**Figure 3:**
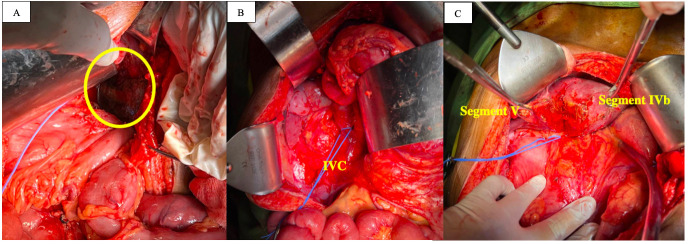
(A) shows the knife in situ. (B) shows no injury to the inferior vena cava (IVC). (C) shows the knife penetrated between segments IVb and V of the liver with 40% parenchymal disruption

**Figure 4:**
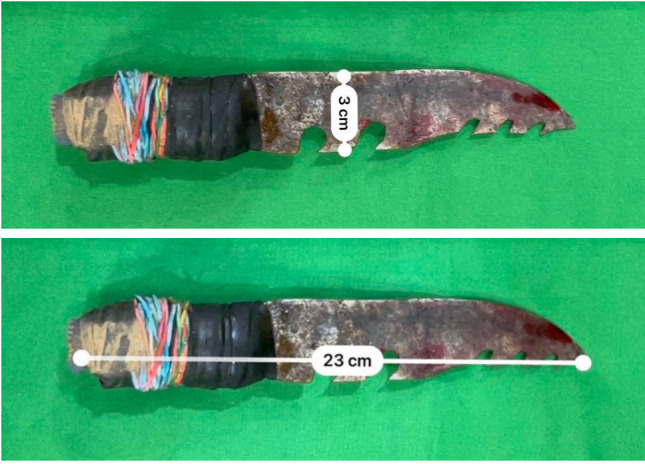
Shows the knife to be serrated with a length of 23 cm, and width of 3 cm.

In our case, no further imaging was performed, and the patient was immediately brought to the operating room. The expertise and surgical collaboration of the trauma surgeon and hepatobiliary surgeon was sought, due to the expected injuries based on the location of the knife-in-situ. The assistant surgeon stabilized the 23cm knife that was inserted into the epigastric area. The approach was via a midline incision, which was elongated from the wound distally for access to the abdominal cavity. Intraoperative findings (figure 3) revealed that the knife (figure 4) penetrated between segments IVb and V of the liver, in which dissection was done along this course. To mobilize and expose the inferior vena cava, the abdominal aorta, and the retroperitoneal structures; Cattell-Braasch and extended K0cher’s maneuver were done. Upon complete mobilization, the inferior vena cava was identified with no injury (figure 3). The tip of the knife was noted to penetrate through the caudate lobe, just 2cm above the inferior vena cava. The knife was removed under direct visualization, by separating the segments IVb amd V of the liver using electrocautery. There was 40% parenchymal disruption with Strasberg A2 bile duct injury with minimal bile leak (figure 3). The bile duct injury was repaired primarily using polypropylene 5-0 in an interrupted fashion, and a single Jackson-Pratt drain was placed. There were no injuries to the vascular structures, bowels, and pancreas.

Postoperatively, the patient was stable and on the 4th postoperative day, liver enzymes were normal and the patient was started on a diet. He was subsequently referred to a psychiatrist for counseling. During the course, the drain was ~100cc serosanguinous which decreased to <10cc sanguineous character, with no bile leak. On the 8th postoperative day, the drain was removed; and the patient improved and was discharged with no further imaging.

In the Philippines, trauma is one of the leading causes of death.^1^ Penetrating abdominal injuries are a common entity, however patients presenting with in situ foreign bodies are rare. There is no current national guideline for the management of abdominal stab wounds (SW) and thus the treatment is largely clinician-dependent. Most literature on the topic consists of anecdotal case reports.^2–4^ Hence, this poses a unique challenge to clinicians due to the multitude of anatomical structures that may be injured. Since in situ injuries are usually removed under direct visualization, the approach to surgery may be difficult. These injuries are particularly difficult to manage in low-resource settings.

In general, in both hemodynamically stable and unstable patients, if the knife is retained in the anterior abdominal wall, a laparotomy is required and is the preferred approach since there is a high positive laparotomy rate in these patients.^5^ A published series of 42 consecutive cases^5^ showed that 72% of all laparotomies performed for anterior abdominal SW with a retained knife had intra-abdominal injuries.

In our case, since the penetrating injury was to the anterior abdomen, we opted to conduct immediate surgical exploration without imaging. This decision aimed to stabilize the weapon in its current position and avoid additional delays, as ultimately imaging would not alter our treatment strategy. The management of self-inflicted penetrating abdominal trauma with a knife in situ is perplexing, frequently necessitating surgical expertise. This particular case underscores the collaborative surgical intervention involving a trauma surgeon and a hepatobiliary surgeon in the safe retrieval of a knife in situ during trauma surgery. For surgeons practicing in both rural and urban settings, the following key learning points can serve as a valuable guide in the management of self-inflicted penetrating abdominal trauma.

### Learning Points:

1. Perform primary and secondary assessments using ATLS principles, and stabilize any life-threatening conditions. If the object is still in place, stabilize the object to prevent further injuries.It is crucial to prevent the uncontrolled removal of the object outside the operating room under any circumstances.

2. If the patient is hemodynamically unstable or if the object is located in the anterior abdomen, perform immediate surgical exploration and extraction of the knife-in-situ under direct visualization.

3. If the patient is hemodynamically stable and the location is in the posterior abdomen, consider performing imaging studies, if available to identify proximity to critical structures since most cases do no require formal laparotomy.
